# Evaluating fractal value of mandibular ramus and condylar volume in patients undergoing orthognathic surgery: a systematic review and meta-analysis

**DOI:** 10.1186/s40902-025-00468-7

**Published:** 2025-07-01

**Authors:** Mehran Rahbar, Ali Sharifi, Javad Hayati Garjan, Mojtaba Sheykhian

**Affiliations:** Department of Oral and Maxillofacial Surgery, Faculty of Dentistry, Tabriz University of Medical Sciences, Tabriz, Islamic Republic of Iran

**Keywords:** Orthognathic Surgery, Mandibular ramus, Condylar volume, Class II malocclusion, Class III malocclusion

## Abstract

**Aim:**

The aim of the present study was to evaluate condylar volume and mandibular ramus in patients undergoing orthognathic surgery.

**Method:**

Relevant keywords were searched in the international databases Cochrane, Embase, and MEDLINE up to February 2025. Study selection criteria were based on the PICOS strategy; randomized clinical trial studies, cohort studies, cross-sectional studies, case–control studies (study (S)) that examined changes in condylar and ramus position (Outcome (O)) in orthognathic surgery as skeletal treatment (Intervention (I)) for Class III versus Class II (Comparison (C)) in patients who had undergone orthognathic surgery (Population (P)). Data were collected based on study findings from three-dimensional (3D) cephalometric/cone-beam computed tomographic (CBCT)analysis and measurements of condylar angle, volume, and position. The methodological index for non-randomized studies (MINORS) used to determine the quality of the studies. Mean differences were used as an effect size with fixed-effects model and inverse-variance methods of 95% confidence intervals (CI). Meta-analysis was performed using Stata (as of version 17).

**Result:**

The mean differences in condylar height between Class II and Class III were 2.19 mm (MD 2.19 mm 95% CI; 1.32 mm, 3.96 mm; *p* < 0.05). The mean differences in ramus angle between Class II and Class III were − 0.02° (MD − 0.02 95% CI − 0.06, 0.03; *p* > 0.05).

**Conclusion:**

Based on the meta-analysis of the present study, orthognathic surgery did not significantly affect the microstructure of the mandibular ramus in the correction of class III malocclusions. In Class II, the condyle height was significantly reduced after orthognathic surgery, while the condyle width did not change.

**Trial registration:**

PROSPERO CRD420251054773.

## Introduction

Numerous functional and cosmetic changes can result from severe malocclusions combined with skeletal abnormalities [1]. Orthognathic surgery (OS) and orthodontic treatment must be carefully combined to correct these deformities [2]. One of the biggest challenges with OS is the proper position of the mandibular ramus in the correct position during osteosynthesis [3]. This is important because the morphological changes in the temporomandibular joint (TMJ) are directly related to the postoperative condylar position, essential for surgical stability [4]. Therefore, maintaining the physiological position of the mandibular ramus and minimizing intraoperative condylar movement remains the most effective means of alleviating postoperative temporomandibular joint problems [5, 6]. Bilateral sagittal osteotomy is the most commonly utilized surgical method for correcting skeletal class II and III discrepancies [7, 8]. OS-induced three-dimensional (3D) shifts in the mandible, maxilla, or both affect the TMJ, the masticatory muscles, the soft tissues around it, and ultimately the symptoms of temporomandibular disorders (TMDs) [9]. Mandibular advancement typically causes the mandibular ramus to move anteriorly within the mandibular fossa, which causes the entire mandibular ramus/temporomandibular disc complex to follow this movement temporarily [10].

Although various methods are used in orthognathic surgery, they all call for osteotomies and fixations. Furthermore, the fixation techniques can significantly impact the condyle’s ultimate position and its relationship to the TMJ [11]. Additionally, there are risks, possible complications, and long-term effects of orthognathic surgery, such as structural condylar changes [11, 12]. Altered load distributions may impact the TMJ, precisely a new condylar position brought about by alterations in one or both maxillary bones [13]. The TMJ may remodel or resorb to adapt, which can cause structural alterations that change the shape of the condylar bone and/or cause temporomandibular joint (DTM) dysfunction [14]. Furthermore, one of the primary causes of long-term skeletal relapse following orthognathic surgery is condylar resorption [15].

Evaluating the post-OS bone remodeling of the mandibular ramus is crucial for tracking the development of joint health and creating successful treatment plans [3]. For accurate clinical monitoring and to anticipate and address possible complications, it is critical to comprehend how the condylar bone structure changes over time following OS [12]. The goal is to maximize clinical results and patient quality of life. Thus, the aim of the present study was to evaluate condylar volume and mandibular ramus in patients undergoing orthognathic surgery.

## Method

### Search strategy and information sources

Based on the study’s objectives, pertinent keywords were searched in the international databases Cochrane, Embase, and MEDLINE until February 2025. Additionally consulted were Google Scholar, CENTRAL (Cochrane Central Register of Controlled Trials), WOS (Web of Science), EBSCO, ISI, Elsevier, and the Scopus Wiley Online Library. The current study was conducted based on the 27-point PRISMA 2020 checklist [16]. The protocol for this study was prospectively registered with the PROSPERO database of systematic reviews (CRD420251054773).

The search strategy used in MEDLINE (via PubMed):

(((((“Orthognathic Surgery”[Mesh]) OR “Orthognathic Surgical Procedures”[Mesh]) OR “Orthognathic Surgery/methods”[Mesh]) AND (“Malocclusion”[Mesh] OR “Malocclusion, Angle Class III”[Mesh] OR “Malocclusion, Angle Class II”[Mesh])) AND “Mandibular Condyle”[Mesh]) AND “Osteotomy, Sagittal Split Ramus”[Mesh].

### Selection criteria

The study was limited to English-language publications. The PICOS strategy was used to answer the questions in the present study. Population (P): Patients undergoing orthognathic surgery; Intervention (I): Orthognathic Surgery as Class III and Class II skeletal treatment; Comparison (C): Class III vs. Class II; Outcome (O): condylar and ramus position changes; study (S): randomized clinical trials, cohort studies, cross-sectional studies, case–control studies. Studies have been conducted in a review, laboratory and animal form; books; qualitative studies; studies with incomplete data and case report studies were excluded from the study.

### The process of selection and data collection

Two blind and independent researchers reviewed the data of the selected studies and the third researcher summarized. The data was collected using a pre-designed form by the research team that includes sections such as the name of the first author of the study, year of publication, number of patients, gender, mean age, malocclusion type, orthognathic surgery, TMJ, and radiographic evaluation.

### Statistical heterogeneity

Chi-square test (*χ*2) and *I*^2^ to determine heterogeneity between studies. The value of *I*^2^ checked in four levels (low heterogeneity ≤ 25%; moderate 25–50%; substantial 50–75%; considerable > 75%.

### Methodological quality

To assess methodological quality, two blinded and independent investigators reviewed the selected studies, and any discrepancies were corrected by the third author. The risk of bias was estimated using the methodological index for non‐randomized studies (MINORS). MINORS included five additional items for comparative analyses and seven for evaluating non-comparative research. There were three possible scores for each item: 0 for not reported, 1 for reported but inadequate, and 2 for reported and appropriate. The highest possible total score for comparative studies is 24, while the maximum score for non-comparative studies is 16 [17].

### Data analysis

Mean differences were used as an effect size, using a fixed-effects model and inverse-variance methods with 95% confidence intervals (CI). Meta-analysis was performed using Stata (as of version 17). Statistical significance was considered less than 0.05.

### Grading of recommendation, assessment, development and evaluation (GRADE) assessment

The assessment of evidence quality was performed according to the Grading of Recommendations Assessment, Development and Evaluation (GRADE) process [18]. A summary of each GRADE domain is provided after the detailed evidence review.

## Result

### Description of studies

**Fig. 1:**
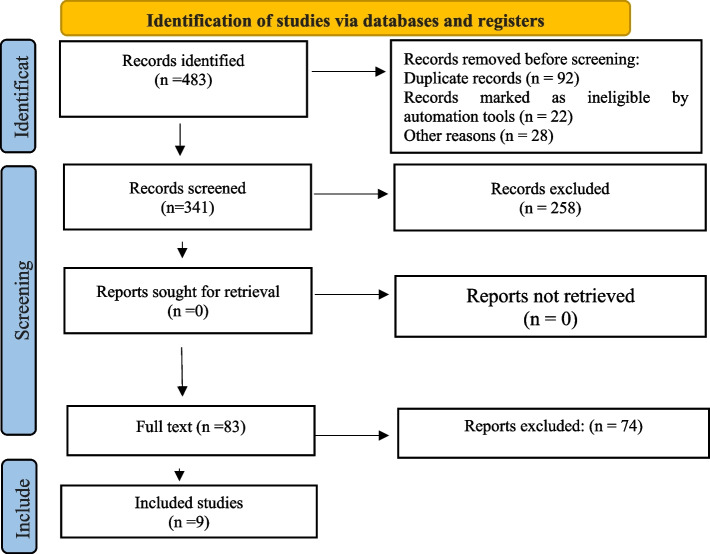
PRISMA 2020 checklist

Four hundred eighty-three articles were found in international databases during the first search using related keywords. Two blind, independent researchers examined the articles, and any that were redundant or had nothing to do with the study’s subject were removed. The full texts of 83 articles were reviewed after the abstract of 341 studies had been assessed against the inclusion criteria (258 articles were excluded at this stage); only nine were included in the study because they aligned with the objectives (Fig. 1).

## Study characteristics

Three hundred ninety-eight patients, 322 women and 76 men, with 23.45 mean age, were included. Malocclusion III, and II were reported in 198 and 200 patients, respectively. The TMJs, including both the condyle and disc, were segregated into two groups, with 256 joints in class II and 256 joints in class III; three studies not reported [3, 19, 20]. The characteristics of the studies are summarized in Table 1.

**Table 1:** Characteristics of studies included for present meta-analysis

Study, years	Study type	Number of condyles	Number of patients		Gender		Mean age	Orthognathic surgery	Analysis	TMJs	
			Class II	Class III	Female	Male				Class II	Class III
Ueki et al. 2025 [21]	R	142	34	37	71	0	24.2	Bilateral SSRO with Le Fort I osteotomy	Cephalometric analysis, MRI assessment, CT measurement	68	74
Guo et al. 2024 [22]	R	64	7	14	16	5	22.4	Orthognathic surgery	Cephalometric analysis, MRI assessment, CT measurement	32	32
Kussaba et al. 2024 [3]	R	–	27	30	38	19	25	Maxillomandibular advancement/mandibular setback surgery	CBCT	–	–
Muftuoglu et al. 2024 [23]	R	23	22	16	23	15	20.4	Mandibular surgery with bilateral sagittal split ramus osteotomy	Panoramic radiographs	22	16
Ueki et al. 2021 [14]	R	92	23	23	46	0	23.5	Bilateral SSRO with modified fixation	Cephalometric analysis, MRI assessment, CT measurement	46	46
Hsu et al. 2021 [19]	R	–	34	17	25	26	26	Orthognathic surgery	CBCT	–	–
Takayam et al. 2019 [24]	Cross-sectional	84	21	21	42	0	NR	Sagittal split ramus osteotomy with Le Fort I osteotomy	Cephalometric analysis, MRI assessment, CT measurement	42	42
Yin Q., 2019 [20]	R	-	11	19	19	11	23	BSSRO	Cephalometric analysis, CT measurement	–	–
Ueki et al. 2018 [25]	R	92	21	21	42	0	23.5	Bi-maxillary surgery	Cephalometric analysis, MRI assessment, CT measurement	46	46

*R* retrospective study

### Bias assessments

According to MINORS, Table 2 shows that six of nine studies were high quality.

**Table 2:** Bias assessments of included studies according to MINORS

Study, years	A	B	C	D	E	F	G	H	I	J	K	L	Score
Ueki et al. 2025 [21]	★	★★	★★	★	☆	★★	★★	★★	☆	★★	★★	★★	18
Guo et al. 2024 [22]	★	★	★★	★★	☆	★★	★	★★	☆	★★	★★	★★	17
Kussaba et al. 2024 [3]	★	★	★★	★	☆	★★	★	★	☆	★★	★★	★★	15
Muftuoglu et al. 2024 [23]	★	★★	★	★	☆	★	★	★	☆	★★	★★	★★	14
Ueki et al. 2021 [14]	★★	★★	★★	★★	☆	★	★★	★★	☆	★★	★★	★★	18
Hsu et al. 2021 [19]	★★	★	★★	★	☆	★	★★	★	☆	★★	★★	★★	16
Takayam et al., 2019 [24]	★	★★	★★	★★	☆	★★	★	★	☆	★★	★★	★★	17
Yin Q., 2019 [20]	★	★	★★	★★	☆	★★	★★	★	☆	★★	★★	★★	17
Ueki et al., 2018 [25]	★	★	★	★	☆	★	★★	★★	☆	★★	★★	★★	15

*A* aim, *B* inclusion of consecutive patients, *C* prospective collection of data, *D* endpoints appropriate to the aim of the study, *E* unbiased assessment of the study endpoint, *F* follow-up period appropriate to the aim of the study, *G* loss to follow up less than 5%, *H* prospective calculation of the sample size, *I* adequate control group, *J* contemporary groups, *K* baseline equivalence of groups, *L* adequate statistical analysis [26]

**Fig. 2:**
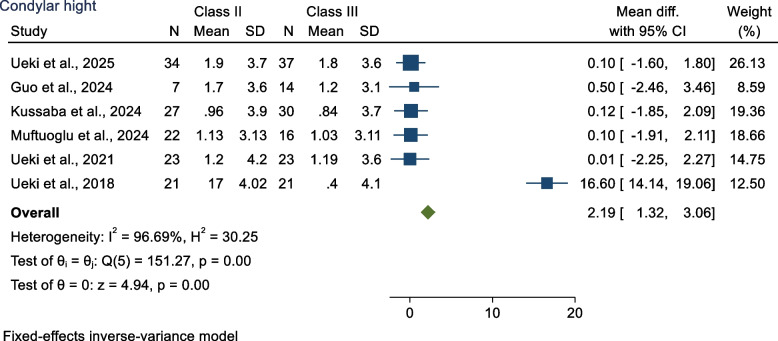
Forest plot showed condylar height for Class II and Class III patients

The mean differences in condylar height between Class II and Class III were 2.19 mm (MD 2.19 mm 95% CI 1.32 mm, 3.96 mm; *p* < 0.05), decreased significantly more in the Class II group than in the Class III group (*p* < 0.05) (Fig. 2). Heterogeneity between studies was estimated to be high according to I^2^ = 96.69% (*p* < 0.001).

**Fig. 3:**
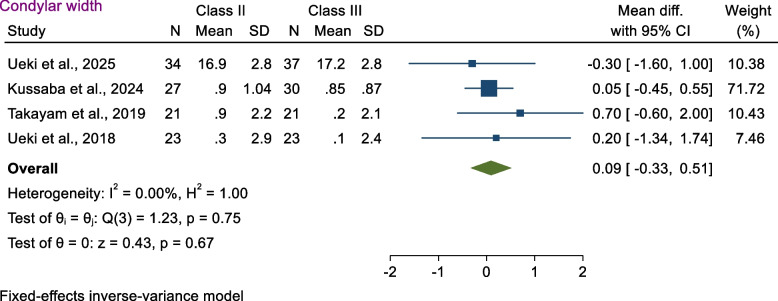
Forest plot showed condylar width for Class II and Class III patients

The mean differences in condylar width between Class II and Class III were 0.09 mm (MD 2.19 mm 95% CI − 0.33 mm, 0.51 mm; *p* > 0.05); no statistically significant differences were found when comparing the condylar width (*p* = 0.67) (Fig. 3). Heterogeneity between studies was estimated to be low according to *I*^2^ = 0% (*p* = 0.75).

**Fig. 4:**
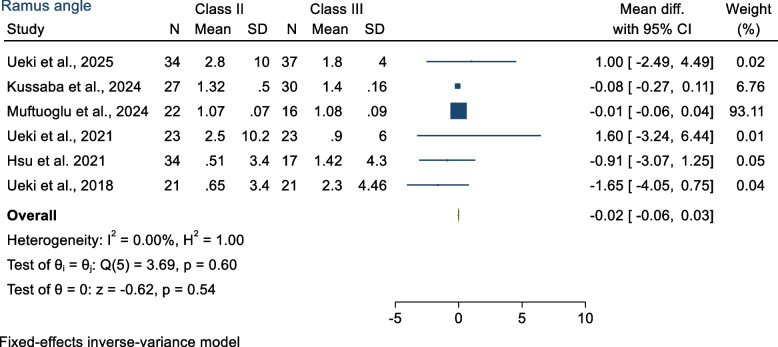
Forest plot showed ramus angle for Class II and Class III patients

The mean differences in ramus angle between Class II and Class III was − 0.02° (MD − 0.02 95% CI − 0.06, 0.03; *p* > 0.05), no statistically significant differences were found when comparing the ramus angle (*p* = 0.60) (Fig. 4). Heterogeneity between studies was estimated low according to *I*^2^ = 0% (*p* = 0.60).

According to Supplement 1, funnel plots showed the relation between a study’s effect size and its precision. Asymmetry in the funnel plot is a sign of publication bias, as it can be seen that there is no publication bias in the present study.

### GRADE assessments

Table 3 shows the certainty of the evidence for each finding.

**Table 3:** GRADE evidence profile

No.. of studies	Certainty assessment					No. of patients		Effect		Certainty
	Risk of bias	Inconsistency	Indirectness	Imprecision	Other	Class III	Class II	Meandifferences(95% CI)		
Condylar height Six retrospective studies	Not serious	Serious	Not serious	Not serious	None	141	134	2.19 mm	Moderate ⨁◯⨁⨁	
Condylar width Three retrospective studies and one cross-sectional study	Not serious	Not serious	Not serious	Not serious	None	111	105	0.09 mm	High ⨁⨁⨁⨁	
Ramus angle Six retrospective studies	Not serious	Not serious	Not serious	Not serious	None	144	161	− 0.02°	High ⨁⨁⨁⨁	

## Discussion

Treatments for Class II and Class III frequently involve bilateral sagittal split ramus osteotomy (BSSO) and sagittal split ramus osteotomy (SSRO) [19]. Despite the many intraoperative advantages, the condylar positional changes are a significant concern. When the mandibular position is altered, the TMJ space changes [27].

Condylar resorption patients typically had a class II morphology, which includes a severe TMJ disorder, a short ramus, a high-angle facial profile, open bite, and mandibular retrognathia [28]. TMJ internal derangement may result from a skeletal pattern featuring a posteriorly rotated ramus and short mandible in individuals with an anterior open bite [29]. However, severe osteoarthritis with anterior disc displacement or condylar resorption has not been reported in patients with skeletal class III [30].

Extensive research on condylar resorption and positional changes after orthognathic surgery has focused on patients with Class II malocclusion [31]. However, the available data poorly support the diagnosis, risk factor identification, and prognosis of condylar resorption in patients with Class III malocclusion.

Condyle resorption is a progressive pathological structural change of the condyle associated with a decrease in volume and/or vertical height [32]. Condyle resorption following orthognathic surgery has an unclear etiology and pathophysiology [33]. Female sex, age (20–30 years), a high lower jaw angle, temporomandibular joint dysfunction, significant surgical mandibular protrusion, counterclockwise rotation of the proximal segment in sagittal cleft osteotomies in the lower jaw, use of improper fixation, and rigid intermaxillary fixation are among the potential risk factors for condyle resorption [30, 34]. Inflammatory arthritis, connective tissue diseases, and autoimmune disorders are common pathological temporomandibular joint abnormalities associated with condylar resorption [35].

The majority of included studies used 3D imaging to measure condylar resorption. In addition, 3D visualization of the condyles using specific protocols and comparable, verified segmentation techniques facilitates volumetric analysis of the condyles [14, 36]. Studies have shown that several measurement techniques influence the morphological changes found in CBCT studies, leading to diverse interpretations. Therefore, knowing the subtle changes associated with condylar reconstruction, two methods have been used: calculating the distance between surfaces and volumetric measurement of the 3D condylar model [19, 37].

Following orthognathic surgery, the present meta-analysis showed no significant change in condylar width and ramus angle between Class II and Class III malocclusion. Condylar height changes were a significant decrease in Class II malocclusion. Studies showed that compared to Class III, Class II experienced more severe morphological condylar changes [14, 19, 25, 38].

Most of the selected studies examined female patients and revealed that condylar resorption was more common in Class II than in Class III, where it was more pronounced. According to these findings, female hormones like 17β-estradiol may have the ability to alter condylar resorption [25, 39]. However, the included studies did not report any sex-dependent differences when a mixed sample was analyzed.

Differences in follow-up period/time of outcome assessment in studies, outcome assessment method (3D or 2D imaging techniques), and small sample size of studies were limitations of the present study. Therefore, studies with similar methodology, larger sample size, and outcome assessment method with 3D imaging are needed to confirm the evidence presented in the present study.

## Conclusion

Based on the meta-analysis of the present study, orthognathic surgery did not significantly affect the microstructure of the mandibular ramus in the correction of class II and class III malocclusions. In class II, the condyle height was significantly reduced after orthognathic surgery, while the condyle width did not change.

The present study shows strong evidence for condylar width and ramus angle; however, due to high heterogeneity between studies in condylar height, these findings should be interpreted with caution and further studies with similar methodology are needed.

## Data Availability

No datasets were generated or analysed during the current study.

## Abbreviations

OS: Orthognathic surgery
TMJ: Temporomandibular joint
DTM: Dysfunction of temporomandibular
3D: Three-dimensional
MINORS: Methodological index for non‐randomized studies
TMDs: Temporomandibular disorders
CI: Confidence intervals
BSSO: Bilateral sagittal split ramus osteotomy
SSRO: Sagittal split ramus osteotomy
